# Detection of a *Serratia sarumanii* outbreak in neonatal intensive care units using SaTScan and whole genome sequencing, Philippines, 2022

**DOI:** 10.5365/wpsar.2026.17.1.1092

**Published:** 2026-01-26

**Authors:** Giselle V Godin, Sonia B Sia, Ferissa B Ablola, June M Gayeta, Marietta L Lagrada, Polle Krystle V Macaranas, Agnettah M Olorosa, Janziel Fiel Palarca, Manuel C Jamoralin, June Janice Borlasa, Ma Fe Laren B Gacho, Rica Marie B Andico, Ida Marrione Q Arriola, Jo-Anne J Lobo, Melanie B Adolfo, Jessica Anne A Dumalag, Joel T Gallardo, Ma Delta S Aguilar, Allyne M Aguelo, Charlotte V Bañes, Genelynne J Beley

**Affiliations:** a Department of Pediatrics, Southern Philippines Medical Center, Davao City, Philippines.; b Antimicrobial Resistance Surveillance Reference Laboratory, Research Institute for Tropical Medicine, Department of Health, Muntinlupa City, Philippines.; c Section of Infectious Diseases, Southern Philippines Medical Center, Davao City, Philippines.; d Department of Pediatrics, College of Medicine, Davao Medical School Foundation, Davao City, Philippines.; e Section of Newborn Medicine, Southern Philippines Medical Center, Davao City, Philippines.; f Department of Biochemistry, College of Medicine, Davao Medical School Foundation, Davao City, Philippines.; * These authors contributed equally.

## Abstract

**Objective:**

This study aimed to demonstrate the benefits of using SaTScan (Boston, MA, USA), a cluster-detection software programme, and whole genome sequencing to investigate a suspected outbreak of *Serratia marcescens* infections in a tertiary government hospital in the southern Philippines. The hospital is part of the national Antimicrobial Resistance Surveillance Program’s network of sentinel sites.

**Methods:**

The investigation followed national outbreak investigation protocols. In May 2022, when evaluation of daily hospital laboratory census data revealed an increase in the number of *Serratia* species in the hospital, an alert was triggered. A concurrent, routine SaTScan analysis of the hospital’s surveillance data by the Antimicrobial Resistance Surveillance Reference Laboratory confirmed a cluster of cases. The Reference Laboratory requested isolates from clinical specimens from the hospital for confirmation of bacterial identification, antimicrobial susceptibility testing and whole genome sequencing.

**Results:**

Six isolates were submitted for genomic analysis, two of which were from the identified cluster. Although originally identified as *S. marcescens*, five of the isolates were subsequently confirmed as *S. sarumanii*. Phylogenetic analysis showed that the two isolates from the cluster were closely related and belonged to the same clade, which may suggest a common source. Three antimicrobial resistance genes were identified, but their phenotypic expression was limited, with one isolate exhibiting resistance mechanisms.

**Discussion:**

This study highlighted the utility of SaTScan for the early detection of potential disease outbreaks. The use of whole genome sequencing enhanced the investigation by enabling the analysis of potential transmission pathways at the genetic level, identification of the outbreak source and the detection of novel species.

*Serratia* species are aerobic, Gram-negative bacilli in the Enterobacteriaceae family that occur naturally in soil and water. Some strains produce prodigiosin, a red pigment, giving colonies a distinctive colouration. (*1*) *Serratia* infections give rise to a wide range of clinical manifestations, (*2*) and outbreaks have led to significant morbidity and mortality, most commonly in patients in intensive care units and most notably in low-birth weight neonates. (*3*) Asymptomatic infections have also been reported. Outbreaks have been traced to contaminated medical equipment, such as bronchoscopes, nebulizers and basins used to collect urine. (*4*) Outbreaks have also been associated with disruptions in infection control techniques caused by overcrowding, understaffing and problems with maintaining nursery routines, particularly hand hygiene. (*5*) In the Philippines, only a few cases have been reported, the most recent of which was in a neonate with a congenital malformation, described by Lappay et al. in 2022. (*6*)

An important characteristic of *Serratia* species is their ability to secrete protein factors such as deoxyribonuclease (or DNase), lipase and haemolysin, which confer resistance to several antibiotics, including cephalosporins, such as cefoxitin and cefotaxime, and penicillins, such as ampicillin and amoxicillin–clavulanic acid. (*7*) Their ability to persist in hospital environments and infect various hosts, coupled with a propensity to act as a reservoir for resistant genes and with evidence of increasing prevalence, means that *Serratia* species pose a significant and growing threat to public health, placing increasing importance on ensuring robust investigations of outbreaks involving these species.

Traditional approaches to detecting and investigating outbreaks of infectious disease have involved demographic case review coupled with laboratory testing of biological specimens from suspected cases or environmental samples, or both, to identify the responsible pathogen. However, phenotypic typing methods often lack the resolution required to accurately pinpoint the source of an outbreak and to trace chains of transmission, especially when isolates cluster together as indistinguishable members of the same organism.

Advances in molecular biology have given rise to genotypic typing methods that overcome some of the limitations of traditional phenotypic methods. Whole genome sequencing (WGS), for example, makes it possible to identify all the genomic characteristics of a bacterium. Moreover, this technique has the capacity to identify functionally important variants in DNA sequences that affect gene expression.

At the same time, SaTScan (Boston, MA, USA), a free-to-access software application available from within WHONET, (*8*) is increasingly being used by epidemiologists globally to detect and describe temporal and spatial clusters of infectious and chronic disease, as well as disease vectors and risk factors. SaTScan can identify whether an infection is randomly distributed over space, over time, or over space and time. Moreover, it allows for evaluation of the statistical significance of disease cluster alarms. (*9*) This combination of technologies – SaTScan and WGS – is emerging as a valuable tool for investigating outbreaks.

In the Philippines, surveillance for antimicrobial resistance (AMR) is the responsibility of the Department of Health. Its Antimicrobial Resistance Surveillance Program operates a network of 28 hospital surveillance sites, located in all 18 administrative regions of the country. Laboratory testing of clinical samples is conducted by the Antimicrobial Resistance Surveillance Reference Laboratory (ARSRL), which is housed at the Research Institute for Tropical Medicine. In 2019, laboratory services were expanded to include WGS for selected isolates, supplementing routine services for bacterial identification and antimicrobial susceptibility testing, and providing enhanced capacity for identifying the genomic characteristics of isolates with emerging AMR. In 2021, as an additional service for the Antimicrobial Resistance Surveillance Program, the ARSRL started to routinely analyse daily microbiological data provided by the programme’s surveillance sites via WHONET, using SaTScan to aid in detecting outbreaks caused by pathogens with potential AMR. Currently, the ARSRL runs SaTScan weekly, and alerts are issued if the software detects groups of bacterial isolates of the same species with a similar resistance profile. Pre-set criteria determine whether any detected clusters are investigated using WGS and assessment of epidemiological data.

On 26 May 2022, the bacteriology department of one of the Antimicrobial Resistance Surveillance Program’s surveillance sites, the Southern Philippines Medical Center, observed an increase in the number of *Serratia* species in the facility. This was promptly communicated to the ARSRL. On 27 May 2022, the Reference Laboratory ran its weekly SaTScan analysis on data routinely submitted by the Medical Center. The SaTScan report confirmed the presence of a potential cluster of *Serratia* species, which met the pre-set criteria for further investigation. The first isolate belonging to this cluster was identified on 10 May 2022.

This study describes the investigation and reporting of the outbreak of *Serratia* infections that occurred in the neonatal intensive care units (NICUs) of the Medical Center. This investigation showcases the strengths of employing SaTScan software in combination with WGS to facilitate prompt outbreak detection and targeted, control-focused responses.

## Methods

### Study design and setting

This investigation of a suspected outbreak of *Serratia* infections at the Medical Center, a sentinel site in the national AMR surveillance programme, used a prospective cohort study design. The Medical Center is a 1200-bed tertiary hospital in Davao City with 70 NICU beds. It is situated in one of the most populous Philippine cities, within an administrative region comprising six cities and 43 municipalities. As an end-referral centre with several highly specialized clinical services, the Medical Center also caters to the health needs of patients from different parts of Mindanao and the country.

### Routine SaTScan analysis

In accordance with protocols for national outbreak investigations, the Data Management Unit of the ARSRL ran its weekly SaTScan analysis in WHONET (*8*, *9*) on 27 May 2022 on daily AMR surveillance data transferred by sentinel sites during the preceding week, initially with the term “resistance profile” as the summary row input. The analysis was then set to run on “resistance profile” with the term “include cluster alerts.” The SaTScan analysis method used was the “space-time permutation model – simulated prospective.” The “maximum cluster length” and “baseline data” fields were set to 100 days and 365 days, respectively, while the “recurrence interval” was set to 365 days and “Monte Carlo simulations” to 9999.

The SaTScan analysis verified the presence of a potential cluster of *Serratia* species isolates exhibiting similar AMR profiles that met pre-set criteria for close spatial, temporal and space-time relationships. (*9*) The identified cluster also met the criteria for further investigation using WGS (**Supplementary Material**). The outcome of the line list of cluster isolates, together with a notification letter, were sent via e-mail to the hospital, addressed to the head of the laboratory and the Infection Prevention and Control Committee.



### Laboratory and environmental analysis

The sentinel site was requested to send the six isolates in the identified cluster to the ARSRL, as well as any environmental samples collected. The Reference Laboratory conducted confirmatory bacterial identification using an automated system (Vitek 2, bioMérieux, Marcy-l’Étoile, France) and conventional biochemical tests.

The isolates included in this study were all tested against nine antibiotics: amikacin, cefepime, cefotaxime, ceftriaxone, ertapenem, gentamicin, imipenem, meropenem and tetracycline. The zone of inhibition and minimum inhibitory concentration were interpreted following the guidelines of the Clinical and Laboratory Standards Institute. (*7*)

### Whole genome sequencing and bioinformatics analysis

DNA was extracted from a single colony in each of the six isolates using a DNA extraction kit (Nexttec Biotechnologie, Hilgertshausen, Germany). DNA libraries were prepared using the Illumina Nextera DNA Flex Library Prep Kit (Illumina, San Diego, CA, USA), which employs bead-linked transposome technology to simultaneously fragment and tag DNA with adaptor sequences. Following tagmentation, a magnetic bead-based clean-up was performed, and indexed primers were used for polymerase chain reaction amplification. The quality and concentration of the amplified libraries were assessed using fluorometric quantification. Pooled libraries were then subjected to sequencing on the Illumina MiSeq platform.

The study used the Bactopia pipeline (v. 3.1) (*10*) for genomic analysis. Genome assembly was performed using Shovill (v. 1.1) (*11*) with SPAdes (v. 4.1; St Petersburg genome assembler), (*12*) targeting a genome size of 5.2 Mb and coverage of 100x. Multilocus sequence typing (MLST) was conducted using MLST software (v. 2.23), which uses PubMLST (*13*) schemes. Phylogenetic analysis employed the Snippy (v. 4.6) (*14*) workflow; the reference genome (US National Library of Medicine, Reference Sequence collection: GCF_002264285.1) was selected based on the closest genome identified by Mash (v. 2.3) (*15*) from the two clustered isolates.

A maximum likelihood phylogenetic tree was generated with IQ-TREE (v. 2.2.2.7) (*16*) using the general time-reversible + invariant sites + γ distribution substitution model, and single nucleotide polymorphism (SNP) distances between isolates were calculated with snp-dists (v. 0.8.2). (*17*) AMR was predicted using AMRFinderPlus (v. 4.0.19), (*18*) with database version 2024–12–18.1, specifying the options -p, -n and -g for *S. marcescens*, and considering AMR determinants and mutations with at least 90% coverage and 90% identity. Raw sequence data were deposited in the United States National Center for Biotechnology Information under project accession identification PRJNA1023302.

## Results

The sentinel site submitted six isolates. Two of these isolates were from the identified cluster; the other four were convenience samples. It was not possible to retrieve and forward other cluster isolates to the Reference Laboratory because the site had discarded them by the time the ARSRL informed it of the clustering of *Serratia* species. Moreover, no environmental sampling had been conducted at the site before or after notification, so no environmental isolates were available for analysis. The results of the genomic characterization and analyses of the six isolates included in this study were concluded by 9 July 2022.

Three of the six isolates were from NICUs 1 and 2; two were from the Medicine Burn Center and one was from Medicine Ward 4 (**Table 1**). The isolates from the two NICU samples were presumed to be nosocomial isolates (i.e. the specimens were collected ≥ 72 hours after admission). The two cluster isolates (22ARS_DMC0256 and 22ARS_DMC0258) and one non-cluster isolate (22ARS_DMC0257) were susceptible to all tested antibiotics. One non-cluster isolate (22ARS_DMC0255) showed resistance to cephalosporins and gentamicin; two non-cluster isolates (22ARS_DMC0259 and 22ARS_DMC0260) demonstrated resistance to tetracycline (**Table 2**).

**Table 1:** Demographic data of patients with *Serratia sarumanii* or *Serratia marcescens* isolates, southern Philippines, 2022 (*n* = 6)

Accession no.	Age	Ward	Specimen type	Admission date	Specimen date
22ARS_DMC0255	1 month	NICU1	Tracheal aspirate	20 April 2022	7 June 2022
22ARS_DMC0256^a^	4 days	NICU1	Tracheal aspirate	20 May 2022	23 May 2022
22ARS_DMC0257	42 years	Medicine Ward 4	Tracheal aspirate	26 May 2022	28 May 2022
22ARS_DMC0258^a^	2 months	NICU2	Tracheal aspirate	23 May 2022	27 May 2022
22ARS_DMC0259	63 years	Medicine Burn Center	Wound	21 May 2022	5 June 2022
22ARS_DMC0260	35 years	Medicine Burn Center	Wound	19 April 2022	3 June 2022

NICU1: neonatal intensive care unit 1; NICU2: neonatal intensive care unit 2.

^a^ Isolates included in the cluster.

**Table 2:** Results of antimicrobial susceptibility testing of *Serratia sarumanii* and *Serratia marcescens* isolates, southern Philippines, 2022 (*n* = 6)

Accession no.	Tetracycline	Cephalosporins			Aminoglycosides		Carbapenems		
		Cefotaxime	Cefepime	Ceftriaxone	Amikacin	Gentamicin	Imipenem	Meropenem	Ertapenem
22ARS_DMC0255	S	R	R	R	S	R	S	S	S
22ARS_DMC0256^a^	S	S	S	S	S	S	S	S	S
22ARS_DMC0257	S	S	S	S	S	S	S	S	S
22ARS_DMC0258^a^	S	S	S	S	S	S	S	S	S
22ARS_DMC0259	R	S	S	S	S	S	S	S	S
22ARS_DMC0260	R	S	S	S	S	S	S	S	S

R: resistant; S: susceptible.

^a^ Isolates included in the cluster.

All isolates were initially molecularly identified as *S. marcescens*. However, ribosomal MLST identified five of the isolates as *S. sarumanii*; the exception was the non-cluster isolate 22ARS_DMC0259, which was confirmed as *S. marcescens* (**Table 3**). PubMLST sequencing further revealed that the two isolates in the cluster (22ARS-DMC0256 and 22ARS-DMC0258) belonged to sequence type 595, whereas the other isolates had distinct sequence types. Additionally, the genome comparator tool in PubMLST generated a phylogenetic tree that grouped the two cluster isolates together with 42 available *S. sarumanii* sequences from China. The differences between these isolates were minimal, with 33–48 SNP differences.

**Table 3:** Antimicrobial resistance genes detected in *Serratia sarumanii* and *Serratia marcescens* isolates, southern Philippines, 2022 (*n* = 6)

Accession no.	Species identification			Gene		
	Bactopia pipeline (*10*)	PubMLST (*13*)	Sequence type	aac(6′)	blaSRT or blaSST	tet_41
22ARS_DMC0255	*S. marcescens*	*S. sarumanii*	521	aac(6′)	*bla* ^SRT^	–
22ARS_DMC0256^a^	*S. marcescens*	*S. sarumanii*	595	aac(6′)	*bla* ^SRT^	–
22ARS_DMC0257	*S. marcescens*	*S. sarumanii*	506	aac(6′)	*bla* ^SRT^	–
22ARS_DMC0258^a^	*S. marcescens*	*S. sarumanii*	595	aac(6′)	*bla* ^SRT^	–
22ARS_DMC0259	*S. marcescens*	*S. marcescens*	1035	aac(6′)	*bla* ^SST^	*tet_41*
22ARS_DMC0260	*S. marcescens*	*S. sarumanii*	406	aac(6′)	*bla* ^SRT^	–

MLST: multilocus sequence typing.

^a^ Isolates included in the cluster.

**Fig. 1:**
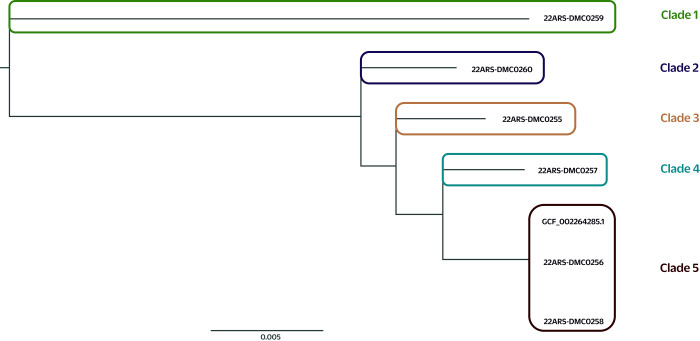
EPhylogenetic tree of Serratia sarumanii and Serratia marcescens isolates, southern Philippines, 2022 (*N* = 6)

Phylogenetic analysis of the sequencing data from the five *S. sarumanii* and one *S. marcescens* isolates showed five distinct clades (**Fig. 1**). The isolate (22ARS-DMC0259) in clade 1 was from a wound sample collected on 5 June 2022 from a 63-year-old male in the Medicine Burn Center. This isolate was considered an outlier, given the high SNP difference between it and the five other isolates (**Fig. 1**, **Table 4**). The second clade comprised isolate 22ARS-DMC0260; it was likewise grown from a wound sample and was collected on 3 June 2022 from a 35-year-old male patient in the Medicine Burn Center. Isolate 22ARS-DMC0255 in clade 3 was from a tracheal sample collected on 7 June 2022 from a 1-month-old patient in NICU 1. Isolates in clades 2 and 3 exhibited distinct antimicrobial susceptibility profiles for cefepime, ceftriaxone and gentamicin (**Table 2**). Furthermore, isolate 22ARS-DMC0259 (clade 2) harboured the tet_41 AMR gene, which was absent in isolate 22ARS-DMC0255 (clade 3) (**Table 3**). Clade 4 included isolate 22ARS-DMC0257 from the tracheal specimen of a 42-year-old patient from Medicine Ward 4.

[**insert** Figure 1]

**Table 4:** Single nucleotide polymorphism analysis of *Serratia sarumanii* and *Serratia marcescens* isolates, southern Philippines, 2022 (*n* = 6)

Accession no.	SNP analysis						
	22ARS-DMC0255	22ARS-DMC0256	22ARS-DMC0257	22ARS-DMC0258	22ARS-DMC0259	22ARS-DMC0260	GCF_002264285.1
22ARS-DMC0255	0	44 625	41 351	45 114	169 474	45 554	45 742
22ARS-DMC0256^a^	44 625	0	36 355	**41**	173 220	50 938	93
22ARS-DMC0257	41 351	36 355	0	36 688	162 457	47 433	37 197
22ARS-DMC0258^a^	45 114	**41**	36 688	0	175 793	51 706	87
22ARS-DMC0259	169 474	173 220	162 457	175 793	0	172 931	177 931
22ARS-DMC0260	45 554	50 938	47 433	51 706	172 931	0	52 439
GCF_002264285.1	45 742	93	37 197	87	177 931	52 439	0

GCF: reference genome; SNP: single nucleotide polymorphism.

^a^ Isolates included in the cluster. The numbers in bold highlight the minimum SNP difference between the two isolates included in the cluster.

The two cluster isolates genotypically belonged to clade 5: 22ARS-DMC0258 was recovered from a tracheal aspirate sample collected on 27 May 2022 from a 2-month-old male in NICU 2, and 22ARS-DMC0256 was recovered from a sample collected on 23 May 2022 from a 4-day-old female in NICU 1. Furthermore, the SNP difference between these two isolates, at 41 units  (**Table 4**), was minimal, and they exhibited identical phenotypic antimicrobial susceptibility testing results.

Three AMR genes were identified across the six *Serratia* isolates (**Table 3**), but their phenotypic expression was limited, with only one isolate exhibiting resistance mechanisms. The aac(6′) gene (aminoglycoside 6′-N-acetyltransferase), which confers resistance to aminoglycosides, was phenotypically expressed only in isolate 22ARS_DMC0255 (**Table 2**). Similarly, the *bla*^SRT^ resistance gene, which confers resistance to cefepime and ceftriaxone, was also expressed only in this same isolate.

## Discussion

In May 2022, the Southern Philippines Medical Center, a sentinel site for AMR, alerted the Philippines ARSRL about a potential outbreak of infections caused by *Serratia* species. Subsequent investigations, involving the tandem use of SaTScan and WGS molecular testing, confirmed the presence of a cluster of *S. marcescens* and *S. sarumanii* at the site. The latter had recently been described as a novel species, based on evidence of phenotypic differences from *S. marcescens*, including differences in colony colour, haemolysis on blood agar and antibiotic susceptibility. (*19*) Most notably, the two species have demonstrably different antimicrobial susceptibility profiles, with *S. sarumanii* generally showing resistance to ampicillin, piperacillin, piperacillin–tazobactam and cefotaxime, while *S. marcescens* is typically susceptible to these.

This study showcases the capability of WGS to differentiate closely related organisms and update species identification. Initial phenotyping of the isolates from the sentinel site identified the pathogen as *S. marcescens*, one of the most common agents involved in hospital-acquired bacterial infections. However, subsequent molecular analyses confirmed that five of the six isolates were *S. sarumanii*. Moreover, WGS was able to provide granular evidence of genetic relatedness among the isolates from the identified cluster. The large SNP differences among the four isolates in clades 1–4 and also between isolates in clades 1–4 and those in clade 5 suggested that isolates from clades 1–4 were genetically distinct from one another and thus unlikely to be from a single source. Conversely, given that the two NICU cluster isolates (22ARS-DMC0256 and 22ARS-DMC0258) were presumed to be nosocomial isolates and exhibited only minimal SNP differences, this pointed to a potential hospital-acquired outbreak of *S. sarumanii* infection. It is also interesting that the SNP differences between the two cluster isolates were less than those reported by an investigation of a confirmed outbreak of *S. marcescens* infections in the NICU of a hospital in Australia (41 vs 48). (*4*) Finally, phylogenetic analysis indicated a likely single transmission source for the two *S. sarumanii* isolates recovered from the patients in NICU 1 and NICU 2.

Antibiotic resistance genes (i.e. specific DNA sequences that confer resistance to antimicrobial agents) are expected to be expressed in the presence of an antibiotic or in the presence of harmful bacterial species. However, not every gene for AMR is necessarily expressed. (*20*) Therefore, it is possible that the acc(6′) gene identified in this study was expressed only in isolate 22ARS_DMC0255 and not in the other five isolates, which were susceptible to both amikacin and gentamicin. Only isolate 22ARS_DMC0255 also showed resistance to cefepime and ceftriaxone, raising the further possibility of non-expression of *bla*^SRT^ in the remaining isolates. Nevertheless, the presence of these AMR genes among these isolates poses a potential public health risk, since the transfer of these genes from one organism to another can be mediated by mobile genetic elements, such as plasmids.

Following detection of the cluster of *S. sarumanii* infections, a multidisciplinary team composed of staff from the Infection Prevention and Control Unit, paediatric infectious diseases specialists and neonatologists was convened to implement immediate control measures to prevent and mitigate further transmission. These measures included targeted cleaning and daily thorough disinfection in the NICUs; environmental swabbing, water analysis and limiting of human traffic in the NICUs; and enhanced monitoring of procedures in the microbiology laboratory, from specimen receipt to result release. Subsequently, multiple meetings were held to review and refine the implementation of these measures.

While this study demonstrated the benefits of using SaTScan and WGS to detect and investigate possible outbreaks of bacterial infections in vulnerable hospital patients, it also identified challenges and potential barriers to wider adoption of these methods for AMR surveillance. While not a major concern in this particular study, occasional operational lapses may cause disruptions to the routine uploading of laboratory data about AMR from surveillance sites to the ARSRL, resulting in delays in detecting and verifying potential clusters using SaTScan. The limited storage capacity for biological samples at sentinel sites is another potential risk, as this can impact the availability of isolates for WGS once a potential outbreak has been identified by SaTScan. In this investigation, only two cluster isolates (out of six) were available for sequencing by the time the site was notified. Nevertheless, in this setting, the inclusion of four non-cluster isolates provided valuable spatial and temporal context for the outbreak investigation itself, while also demonstrating the value of genomic analysis in distinguishing a potential outbreak from a coincidental increase in cases. However, the unavailability of environmental samples and swabs from staff was a noted limitation of this investigation, as these may have helped pinpoint the source of the outbreak. From a broader operational perspective, the cost of performing WGS is high, limiting its application in routine purposes, such as surveillance. Sequencing services in many low- and middle-income countries will likely be limited due to constraints on resources and access to hard-to-procure reagents.

### Conclusions

This study highlights the potential advantages of combining WHONET’s SaTScan feature with WGS for detecting and investigating outbreaks of potentially resistant bacterial infections in health-care facilities. In this case, the use of SaTScan prompted a timely investigation of an increase in *Serratia* infections detected in routine laboratory census data, while WGS provided granular evidence of the genetic relatedness of two cluster isolates, indicating that the infections may have come from a single source within the hospital’s NICUs. The use of such tools has the potential to assist in delivering a more focused and efficient response by providing insights for effective outbreak management and resource optimization in health-care settings.
