# Supplementary material for: Detection of a Serratia sarumanii outbreak in neonatal intensive care units using SaTScan and whole genome sequencing, Philippines, 2022
Source: Western Pac Surveill Response J. 2026 Jan 26;17(1):1–9. doi: 10.5365/wpsar.2026.17.1.1092 (PMC12912848; doi:10.5365/wpsar.2026.17.1.1092)
Supplement: Supplementary file 1 [file wpsar-17-1092-s001.pdf]

Supplementary Material. **Criteria for using whole genome sequencing after a cluster was detected by SaTScan analysis of isolates, Antimicrobial Resistance Surveillance Reference Laboratory, Research Institute for Tropical Medicine, Department of Health, Philippines**

A. Should meet the following primary criteria:

a.1. The isolates are target organisms from specimen sources based on basic laboratory procedures in clinical bacteriology, according to WHO and the Clinical Laboratory Standards Institute.[7]

a.1.1. For isolates from urine and respiratory specimens, there should be a sufficient level of growth on the culture to be considered the causative agent of infection.

a.2. At least one epidemiological data point shows a potential transmission pattern, such as:

a.2.1. close isolation times

a.2.2. overlapping locations

B. Must include at least one of the following secondary criteria:

b.1. Involves a vulnerable population

b.1.1. neonates, infants or children <5 years

b.1.2. elderly people

b.1.3. patients in an intensive care unit

b.2. Agents of foodborne and waterborne diseases

b.2.1. *Salmonella* Typhi

b.2.2. invasive non-typhodial *Salmonella*

b.2.3. *Shigella* spp.

b.2.4. *Vibrio cholerae*

b.3. Isolates have the following resistance profiles:

Organism or organism group	Antimicrobial agents and resistance phenotype
Enterobacterales	• XDR<br>• Carbapenem R<br>• Colistin or any other polymyxin
Salmonella and *Shigella* spp.	• Cefotaxime R<br>• Ceftriaxone R<br>• Ciprofloxacin R<br>• Azithromycin R
Acinetobacter baumannii	• Presumptive pan-resistant<br>• Carbapenem R<br>• Colistin or other polymyxin
Pseudomonas aeruginosa	• Presumptive pan-resistant<br>• Ceftolozane–tazobactam I or R<br>• Carbapenem R<br>• Colistin or other polymyxin
Haemophilus influenzae	• Ceftolozane–tazobactam NS<br>• Cefotaxime NS<br>• Ceftazidime NS<br>• Ceftriaxone NS<br>• Carbapenem NS<br>• Any fluoroquinolone NS<br>• Lefamulin NS
Neisseria gonorrhoeae	• Ceftriaxone NS<br>• Cefixime NS<br>• Azithromycin NS
Enterococcus spp.	• Vancomycin R (for Enterococcus faecalis only)<br>• Daptomycin SDD, I or R<br>• Linezolid R
Staphylococcus aureus	• Ceftaroline SDD or R<br>• Vancomycin R<br>• Daptomycin NS<br>• Linezolid R

I: intermediate; NS: non-susceptible; R: resistant; SDD: susceptible–dose dependent; XDR: extensively drug-resistant.

b.4. Isolates included in the cluster are from presumptive nosocomial infections (with specimen date of ≥72 hours from admission)

b.5. Invasive isolate

In view of limited resources, for criteria B.b.1 and B.b.4, the use of whole genome sequencing to investigate potential outbreaks or clustering of isolates will be done only for clusters involving isolates with the following characteristics:

1. isolates are resistant to at least two antibiotic classes in the primary testing panel;
2. isolates are resistant to at least three antibiotic classes, regardless of panel grouping; and
3. isolates were from sterile samples.

In the interim, clusters not meeting any of the prioritization criteria will not be investigated using whole genome sequencing.
